# Improving Skin Color Diversity in Cancer Detection: Deep Learning Approach

**DOI:** 10.2196/39143

**Published:** 2022-08-19

**Authors:** Eman Rezk, Mohamed Eltorki, Wael El-Dakhakhni

**Affiliations:** 1 School of Computational Science and Engineering McMaster University Hamilton, ON Canada; 2 Faculty of Health Sciences McMaster University Hamilton, ON Canada

**Keywords:** deep learning, neural network, machine learning, algorithm, artificial intelligence, skin tone diversity, data augmentation, skin cancer diagnosis, generalizability, skin, cancer, diagnosis, diagnostic, imaging, dermatology, digital health, image generation, generated image, computer-generated, lesion

## Abstract

**Background:**

The lack of dark skin images in pathologic skin lesions in dermatology resources hinders the accurate diagnosis of skin lesions in people of color. Artificial intelligence applications have further disadvantaged people of color because those applications are mainly trained with light skin color images.

**Objective:**

The aim of this study is to develop a deep learning approach that generates realistic images of darker skin colors to improve dermatology data diversity for various malignant and benign lesions.

**Methods:**

We collected skin clinical images for common malignant and benign skin conditions from DermNet NZ, the International Skin Imaging Collaboration, and Dermatology Atlas. Two deep learning methods, style transfer (ST) and deep blending (DB), were utilized to generate images with darker skin colors using the lighter skin images. The generated images were evaluated quantitively and qualitatively. Furthermore, a convolutional neural network (CNN) was trained using the generated images to assess the latter’s effect on skin lesion classification accuracy.

**Results:**

Image quality assessment showed that the ST method outperformed DB, as the former achieved a lower loss of realism score of 0.23 (95% CI 0.19-0.27) compared to 0.63 (95% CI 0.59-0.67) for the DB method. In addition, ST achieved a higher disease presentation with a similarity score of 0.44 (95% CI 0.40-0.49) compared to 0.17 (95% CI 0.14-0.21) for the DB method. The qualitative assessment completed on masked participants indicated that ST-generated images exhibited high realism, whereby 62.2% (1511/2430) of the votes for the generated images were classified as real. Eight dermatologists correctly diagnosed the lesions in the generated images with an average rate of 0.75 (360 correct diagnoses out of 480) for several malignant and benign lesions. Finally, the classification accuracy and the area under the curve (AUC) of the model when considering the generated images were 0.76 (95% CI 0.72-0.79) and 0.72 (95% CI 0.67-0.77), respectively, compared to the accuracy of 0.56 (95% CI 0.52-0.60) and AUC of 0.63 (95% CI 0.58-0.68) for the model without considering the generated images.

**Conclusions:**

Deep learning approaches can generate realistic skin lesion images that improve the skin color diversity of dermatology atlases. The diversified image bank, utilized herein to train a CNN, demonstrates the potential of developing generalizable artificial intelligence skin cancer diagnosis applications.

**International Registered Report Identifier (IRRID):**

RR2-10.2196/34896

## Introduction

The “white lens” phenomenon has led to the underrepresentation of dark skin pathology images in dermatology resources [1]. A recent analysis of several dermatology textbooks utilized to educate dermatologists showed that dark skin images represent merely 4% to 18% of the total number of images [2]. As a result, it is challenging for dermatologists to properly diagnose and treat skin pathology in people of color.

Applications utilizing artificial intelligence (AI) have been developing at a rapid pace to aid clinicians in making diagnoses [3,4]. Deep learning (DL), a branch of AI, has been widely employed to develop models as accurate as specialist dermatologists in diagnosing skin cancer [5-8] and common skin conditions [9-12]. However, a major drawback facing the mainstream adoption of DL applications in dermatology is the paucity of training data diversity leading to nonrobust models [13,14].

Han et al [15] developed a DL model to diagnose malignant and benign skin lesions using clinical images. According to their results, the performance of the model was highly dependent on the diversity of the training data. Thus, DL models trained on data with a certain skin color range could not be generalized when tested on data collected from a different population [16]. Rahman et al [17] utilized International Skin Imaging Collaboration (ISIC) images to train and test 5 DL models to diagnose various malignant and benign skin lesions [18]. The models achieved a recall of 88%, 89%, 91%, 88%, and 84%, respectively, and the performance was further boosted by developing an ensemble of the implemented models that achieved a recall of 94%. ISIC images were also utilized to develop a DL framework, DermoExpert [19], to classify up to 7 malignant and benign skin lesions. The framework was trained and tested on ISIC-2016, ISIC-2017, and ISIC-2018 images and achieved an AUC of 0.96, 0.95, and 0.97 for the 3 data sets, respectively.

Although ISIC provides a large publicly available skin images archive, the images were mainly collected from the United States, Europe, and Australia [13], where light skin colors are dominant. This was also confirmed by Kinyanjui et al [20], who studied the skin tone distribution of ISIC images and showed that the skin tone of the images primarily ranged from very light to intermediate. Thus, the aforementioned models trained and tested on ISIC images are not expected to be generalizable to darker skin colors.

Motivated by this necessity, we proposed an algorithm development and validation protocol to perform skin cancer early detection for all skin colors [21]. In the protocol, we considered clinical images to develop the model because clinical images are easy to obtain, unlike dermoscopic images that require a specialist and microscopy. In this paper, we discuss the development and initial internal validation of skin image generation for underrepresented skin colors in publicly available data sets (Phases 2 and 3 of the protocol). This paper aims to (1) generate realistic images with malignant and benign skin lesions using 2 deep learning methods, (2) extensively evaluate the generated images using quantitative ratings as well as qualitative human expert and nonexpert ratings, and (3) develop a preliminary classifier, trained with the generated images, to categorize the images as malignant or benign and to study the generated images’ effect on the classification accuracy.

The remaining article is organized as follows: the methods section explains the materials and techniques utilized to generate and evaluate the images. The subsequent section shows the experimental results of all components involved in this work, and the final section highlights our work limitations, discusses the proposed work in comparison with other existing studies, and concludes our work.

## Methods

### Background

In this work, we implement 2 phases of our ongoing study that aims at leveraging deep learning to improve skin color diversity and thus malignancy detection in any skin color using clinical images. The first phase of our study [21] focused on quantifying the underrepresentation of darker skin colors in dermatology atlases by developing a skin tone categorization tool. The second and third phases of the study, implemented herein, aim to generate images with darker skin color, extensively assess the generated images using several evaluation metrics, and study the impact of the generated images on malignancy detection by developing a classification model trained on the generated images. Finally, the fourth phase, expected to be completed by the end of 2022, will focus on developing an accurate malignancy detection classification model. This model will compile the generated images with text descriptions of skin cancer clinical presentations in darker skin colors and use novel deep learning architectures and ensemble learning approaches to improve classification accuracy. In this section, we explain the characteristics of the utilized data, the image generation methods, and the evaluation techniques employed to achieve the objectives of Phases 2 and 3.

### Study Data Set

We collected 1701 clinical images representing several malignant and benign skin lesions from the publicly available skin image repositories DermNet NZ (994 images) [22], ISIC-2018 JID editorial images (100 images) [17], and Dermatology Atlas (607 images) [23]. Images from DermNet NZ and ISIC (1094 images), referred to as set A, were utilized for generating images, training, and validating the classifier. Meanwhile, Dermatology Atlas images (607 images), referred to as set B, were utilized to test the classifier. The distribution of the data as malignant and benign is listed in Table 1.

The skin tone diversity of the study data sets was investigated using our skin tone categorization tool [21]. The results, summarized in Table 2, showed that the majority (84.1%, n=920) of set A images were categorized as light and intermediate skin tones, while set B was more diverse and had varying skin tone distributions. Based on this, set B will facilitate our evaluation of the generalizability of the classification model developed using the generated images, as it has variant skin tone distribution compared to the training data.

**Table 1:** Study data sets for malignant and benign class distribution [21]. Set A (n=1094): training and validation set; set B (n=607): testing set.

Tumor type	Set A, n (%)	Set B, n (%)
Malignant	634 (58)	508 (83.7)
Benign	460 (42)	99 (16.3)

**Table 2:** Skin tone distribution of the study data sets. Set A (n=1094): training and validation set; set B (n=607): testing set.

Skin tone	Set A, n (%)	Set B, n (%)
Light	690 (63.1)	133 (21.9)
Intermediate	230 (21.0)	198 (32.6)
Tan	110 (10.1)	131 (21.6)
Brown	62 (5.7)	134 (22.1)
Black	2 (0.18)	11 (1.8)

### Image Generation

#### Style Transfer

**Figure 1:**
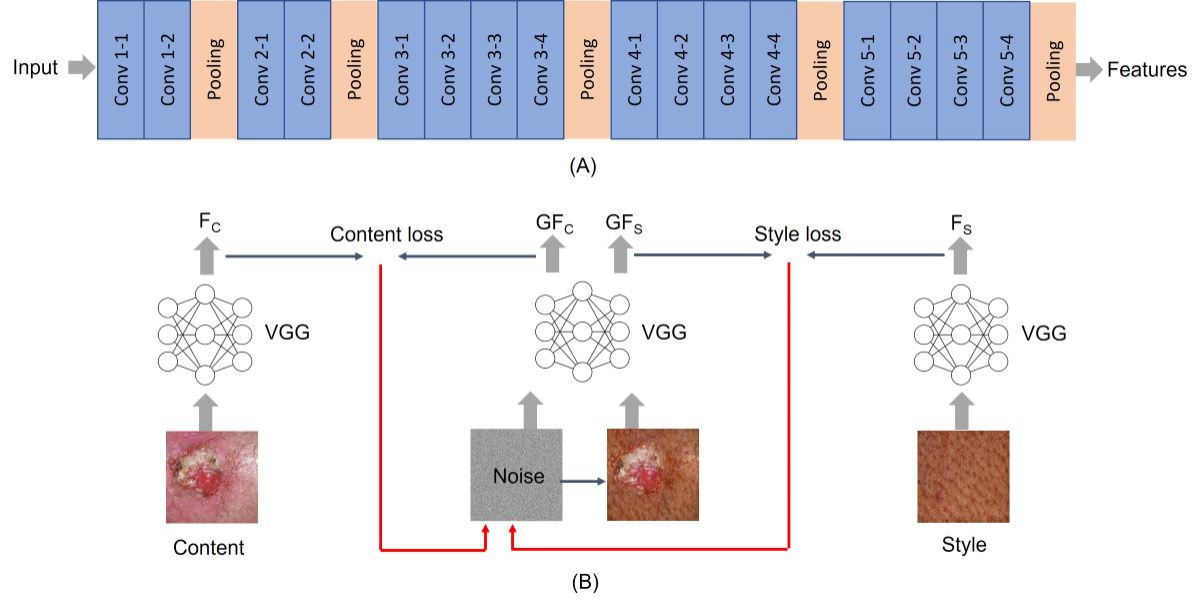
Style transfer (ST) in skin images. (A) VGG architecture. (B) Process of ST.

Style transfer (ST) [24] is an image generation technique developed based on the visual geometry group (VGG)-19 network architecture and trained on the ImageNet database with millions of images [25]. ST utilizes 16 convolutional layers (Conv), 5 average pooling, and no fully connected layers of the VGG-19 architecture, as illustrated in Figure 1A. The ST method, as demonstrated in Figure 1B, primarily works by extracting features from content and style images denoted as F_C_ and F_S_. Then, it iteratively blends the features to generate a new image with content and style features (GF_C_, GF_S_). The content and style losses are calculated as the difference between the original (F_C_, GF_C_) and the generated features (F_S_, GF_S_). The total loss is backpropagated to the VGG network to improve the quality of the generated image.

Since convolutional neural networks (CNNs) trained with an adequate number of annotated data on object recognition can extract high-level features from images independent of their content [26], the ST method can be generalized for feature extraction from skin lesion images. Therefore, ST can be utilized to generate darker skin images without retraining the VGG network. ST was utilized in this work by extracting the features of a light skin image containing the skin pathology and a style image with the target skin color. A new image containing an optimized blend of both feature sets was subsequently generated, starting from a noise image and iteratively improving by minimizing the total loss, as illustrated in Figure 1B. The fine-tuning details of the ST method are discussed in Multimedia Appendix 1.

#### Deep Blending

Deep blending (DB) is an integration of ST and Poisson image blending methods [27], wherein the object of interest from a content image is transferred to the style image while minimizing the sharp intensity and texture change between the content and style images [28]. As in ST, DB utilizes the VGG network to extract the features of the input images and iteratively updates the output image using the calculated loss functions. However, DB works only on the object of interest from the content image and thus requires a segmented object. Moreover, DB essentially works on the blending region where the content object meets the style image. Therefore, DB utilizes 3 loss functions: (1) Poisson-based gradient loss to minimize the change of the blending region gradient, (2) content loss to ensure the semantic of the blending region is similar to the content object, and (3) style loss to ensure the texture of the blending region is similar to the style image. Finally, DB performs 2 rounds of blending; the first round employs the content object and the style image, and the second employs the output blended image of the first round and the style image. The fine-tuning details of the DB method are discussed in Multimedia Appendix 1.

#### Target Skin Color Selection

The target skin color is the style needed to synthesize images in ST and DB methods. To generate images for the underrepresented skin colors in set A, tan, brown, and black skin colors were selected. The selection of the target style images was determined using the individual typology angle (ITA) calculated from the input transformed images [29]. Consequently, the angle was mapped to a skin class according to predefined ITA ranges [30]. The ITA calculation and mapping are explained in Multimedia Appendix 2.

**Figure 2:**
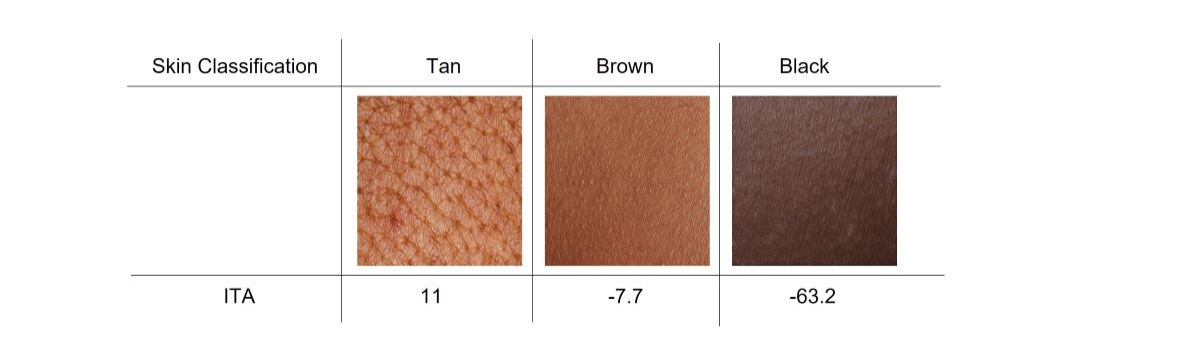
Skin tone classification. ITA: individual typology angle.

Figure 2 shows the selected skin images, to be utilized as style images, with the ITA score and skin classification. The tan skin image was obtained from Dermatology Atlas [23], while the brown and dark skin images were obtained from ShutterStock [31] through a standard license.

### Evaluation

#### Quantitative Evaluation

The quantitative evaluation was performed using the blind referenceless image spatial quality evaluator (BRISQUE) and the structural similarity index measure (SSIM) to assess realism and disease presentation, respectively. BRISQUE is a referenceless metric that quantifies the loss of image realism in the presence of distortions solely using the image being assessed [32]. This method assigns a quality score to each image that correlates well with human quality judgment [32]. The BRISQUE evaluation method is based on 2 main concepts: (1) real images maintain regular statistical properties, and (2) normalized brightness coefficients of a real image approximately follow a Gaussian distribution. As such, image distortion can be captured by a change in the expected statistical properties or deviation from a Gaussian distribution (such as the generalized Gaussian distribution [33] and the asymmetric generalized Gaussian distribution [34], as explained in Multimedia Appendix 3).

The second metric, SSIM, compares the structure, texture, and edges of a reference image with a modified image and provides a similarity score [35]. SSIM was previously used to evaluate the quality of the generated skin lesion images [36]; therefore, SSIM is employed in this study to evaluate the similarity of the generated images with the content image including the disease to measure disease presentation. The SSIM calculation is explained in Multimedia Appendix 3.

#### Qualitative Evaluation

For the qualitative assessment, 62 individuals with varying backgrounds participated in evaluating the generated images. Of the 62 participating individuals, 41 (66.1%) had no medical background and 21 (33.9%) were medical personnel that included 10 (47.6%) attending physicians, 2 (9.5%) physicians in training, 1 (4.8%) nurse, and 8 (38.1%) dermatologists. The first task was a human visual Turing test (VTT), wherein participants (with and without a medical background) were asked to classify the images as real or generated. The responses of the VTT were analyzed to (1) determine the significance of background (medical versus nonmedical personnel) and experience in discovering the generated images and (2) estimate the quality of the generated images by calculating the classification accuracy, false positive rate (FPR), defined as the ratio of generated images classified as real, and true positive rate (TPR), defined as the ratio of real images classified as real.

The second task was a disease identification test carried out solely by dermatologists with varying experience levels. The responses to this test were analyzed to measure the recall (ratio of correctly diagnosed images by dermatologists) of the real and generated images. The 95% CI was calculated using the Clopper-Pearson method [37] to estimate the uncertainty of the reported results.

#### Preliminary Classification Evaluation

**Figure 3:**
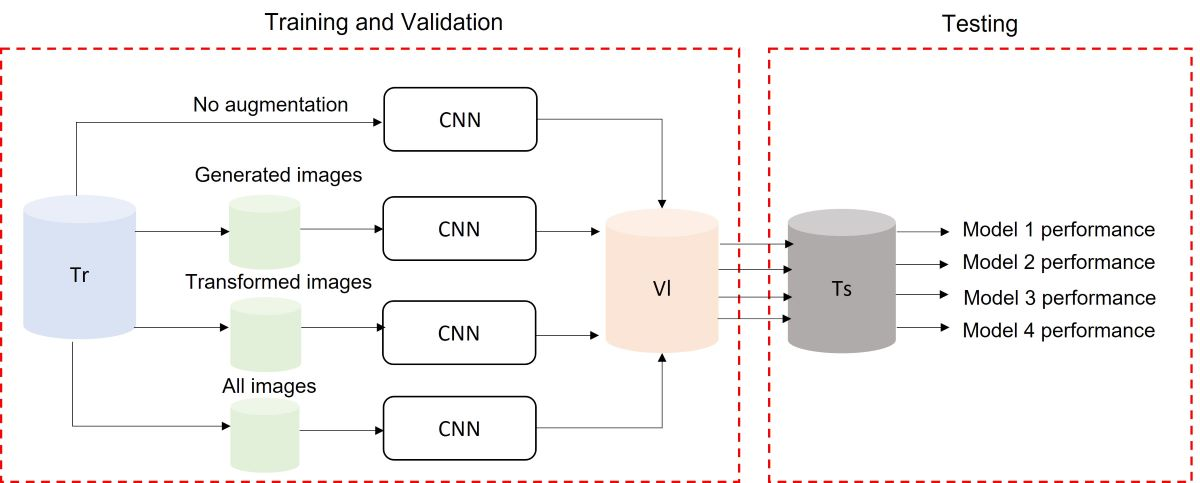
Image classification process. CNN: convolutional neural network; Tr: training set; Ts: test set; Vl: validation set.

To study the effect of the generated images on skin color diversity, the generated images were used to augment the original images of set A to train a CNN and classify the image as malignant or benign. The 1094 images of set A were randomly split, with 80% (n=875) used for training the network and 20% (n=219) used for validation. The CNN training followed 4 data utilization approaches, as illustrated in Figure 3: (a) use the images directly for training without performing any augmentation; (b) augment the images with their corresponding generated tan, brown, and black images; (c) augment the images through geometric transformations, such as flipping, rotating, and adding noise [38]; and (d) augment the images with the generated and transformed images. All models were validated on the same validation set (219 images) and evaluated using separate test data, set B, which included 607 real images with diverse skin tone distribution, as illustrated in Table 2.

**Figure 4:**
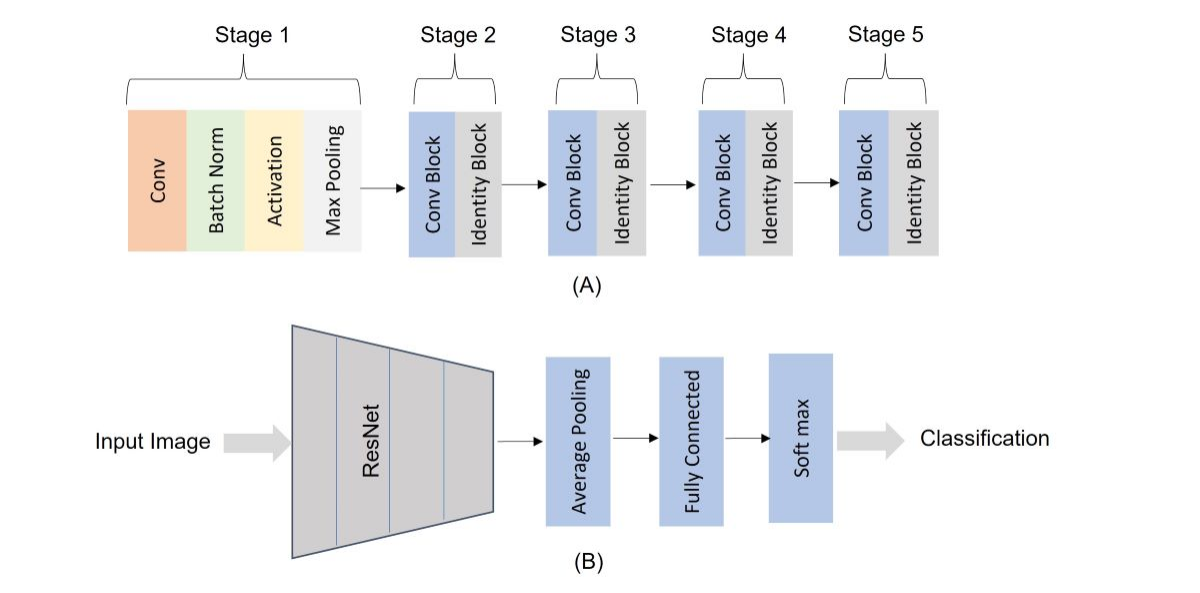
Classification network. (A) ResNet-50 architecture and (B) the customized ResNet-50.

ResNet-50 [39] pretrained on ImageNet images was utilized in our work due to its applicability to dermatology diagnostic tasks [40,41]. The ResNet-50 architecture consists of the 5 stages shown in Figure 4A. For skin lesion classification, we customized ResNet-50 by adding an average pooling layer, a fully connected layer, and SoftMax to classify the lesions as malignant or benign, as shown in Figure 4B. Transfer learning was applied when training the ResNet-50, wherein we froze the first 4 blocks of the ResNet-50 to make use of the ImageNet’s gained weights and trained the last block with the newly added layers to gain new weights. The customized ResNet-50 was trained for 30 epochs and optimized using an Adam optimizer [42] with a learning rate of 0.001. The learning rate was incrementally reduced when there was no improvement in the validation accuracy for 5 consecutive epochs to allow the models to learn more optimal weights [43].

### Ethics Approval

All images utilized in our work were collected from publicly available deidentified data sets. Therefore, we do not require ethics approval.

## Results

**Implementation Details**

All the developed models were implemented on Google Collaboratory Pro with a NVIDIA Tesla P100 GPU. We used Keras [44] with Tensorflow [45] to develop and optimize the models. The average time to generate a single image using the ST method was 46 seconds and 9 minutes using the DB method (performing 2 rounds of image optimization). The time for training the classification models varied based on the data utilization approach; the average training time was 14, 34, 34, and 47 minutes for the no augmentation, generated image augmentation, transformed image augmentation, and all images augmentation, respectively (Figure 3).

### Quantitative Evaluation

Based on the skin tone analysis of the study data set, the 920 images categorized as light (690) and intermediate (230) skin colors were utilized as content, and 2760 images were generated using each method for the tan, brown, and dark style images. Tables 3 and 4 report the average normalized BRISQUE and average SSIM scores for each skin color using ST and DB generation methods, respectively. As the BRISQUE measured the loss of realism in the generated images, lower BRISQUE scores indicated higher realism. As the SSIM measured the similarity between the generated images and the content images, higher SSIM scores indicated a higher similarity to the image including the disease.

It can be seen that the ST method outperformed the DB method in terms of realism by achieving significantly lower average BRISQUE scores in all skin tones (Table 3). The overall BRISQUE score of the ST method was 0.23 (95% CI 0.19-0.27) compared to the DB score of 0.63 (95% CI 0.59-0.67). In terms of disease presentation, ST achieved higher average SSIM scores in all skin tones (Table 4). The overall SSIM score of the ST method was 0.44 (95% CI 0.40-0.49) compared to 0.17 (0.95% CI 0.14-0.21) for the DB method. Across the different tones, there was a consistent change in the BRISQUE metric for both methods resulting from the quality variation of the utilized style images. Similarly, the SSIM changed across skin colors, decreasing for ST and DB for darker colors due to the deviation from the light skin color of the content images.

**Figure 5:**
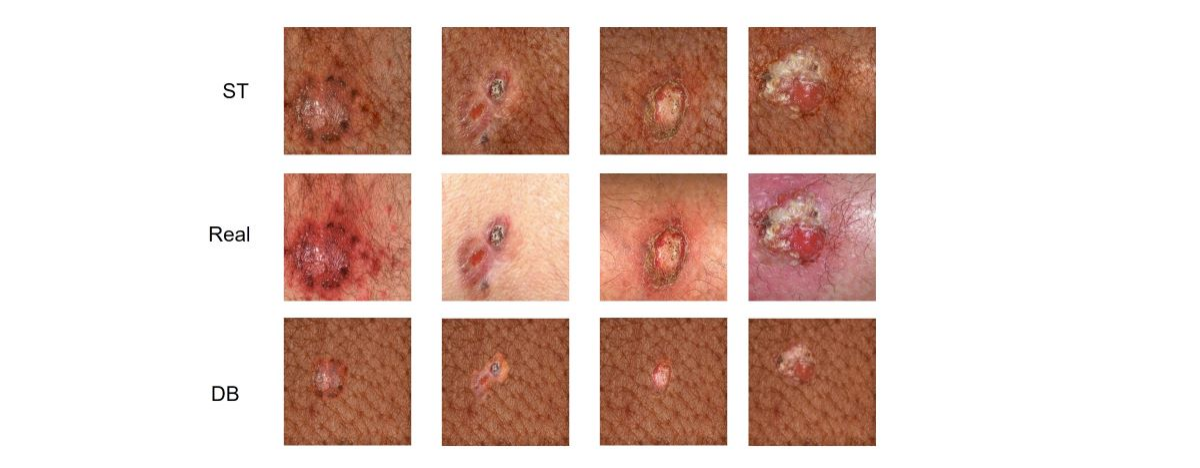
Generated images using style transfer (ST) and deep blending (DB) compared to the real images.

A visual qualitative comparison between the images generated by the ST and DB methods with respect to the real images is demonstrated in Figure 5. The ST-generated images showed clear disease presentation while adding up the pigmentation on the lesion region to match the darker skin color. However, the DB-generated images included the disease region from the content image and focused only on blending the border of the disease with the style image. Therefore, the ST-generated images looked more realistic compared to the DB-generated images.

**Table 3:** Average normalized blind referenceless image spatial quality evaluator (BRISQUE) scores of the style transfer (ST) and deep blending (DB) methods.

Method	Tan	Brown	Black
ST^a^	0.13 (95% CI 0.08-0.19)	0.35 (95% CI 0.27-0.42)	0.22 (95% CI 0.15-0.29)
DB^b^	0.55 (95% CI 0.47-0.63)	0.93 (95% CI 0.89-0.97)	0.42 (95% CI 0.34-0.49)

**^a^**ST: style transfer.

^b^DB: deep blending.

**Table 4:** Average structural similarity index measure (SSIM) scores of the style transfer (ST) and deep blending (DB) methods.

Method	Tan	Brown	Black
ST^a^	0.51 (95% CI 0.43-0.59)	0.44 (95% CI 0.36-0.52)	0.37 (95% CI 0.30-0.45)
DB^b^	0.20 (95% CI 0.14-0.26)	0.17 (95% CI 0.11-0.23)	0.15 (95% CI 0.09-0.21)

**^a^**ST: style transfer.

^b^DB: deep blending.

### Qualitative Evaluation

For the human qualitative evaluation component, we conducted 2 assessments, a VTT to evaluate the realism of the generated images and a disease identification assessment to evaluate disease presentation. As the ST method showed superior quantitative evaluation compared to DB, we conducted all human evaluations on the ST images.

The human VTT was performed on 45 real and 45 generated images to evaluate realism. A total of 54 participants, including 41 (75.9%) without a medical background and 13 (24.1%) medical personnel, including 10 (76.9%) attending physicians, 2 (15.4%) physicians in training, and 1 (7.7%) nurse, were asked to classify the images either as real or generated. First, we analyzed the scores of each participant to study the significance of the background and years of experience in identifying the generated images correctly. The generated score (number of generated images correctly identified) was set as the outcome, and the real score (number of real images correctly identified), background (medical versus nonmedical personnel), and years of experience (0: nonmedical personnel, 1: medical personnel with 2 to 5 years of experience, 2: medical personnel with 6 to 10 years of experience, and 3: medical personnel with more than 10 years of experience) were predictors.

**Figure 6:**
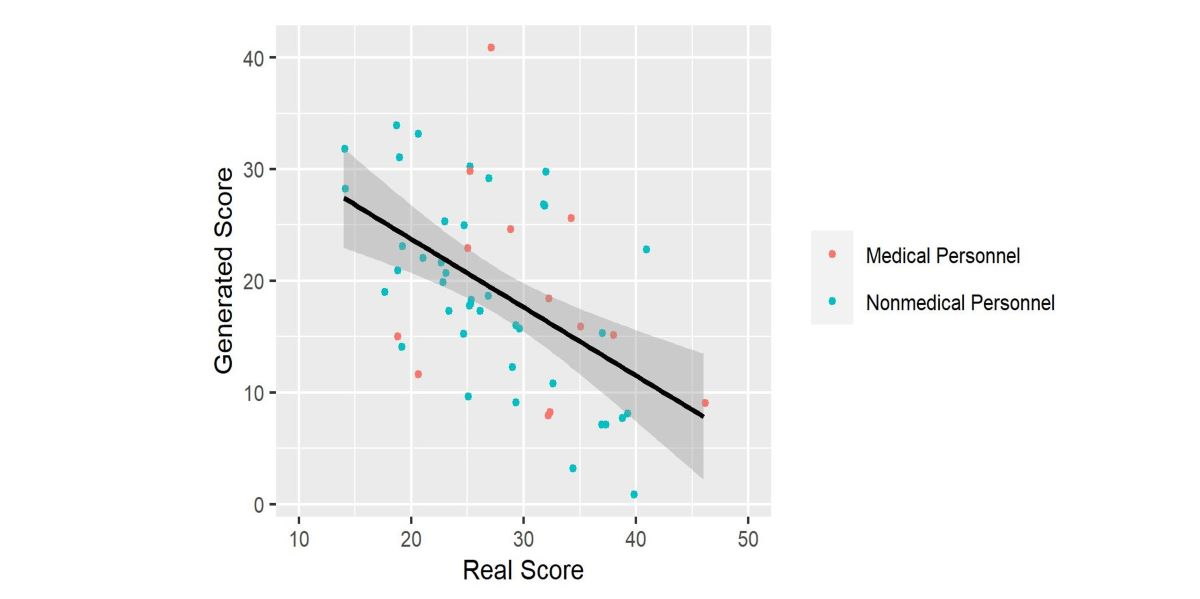
Generated score versus the real score. Line represents the linear regression model with the standard error shaded.

Linear regression was utilized to investigate the significance of the predictors on the outcome. First, the generated score was modeled using the background only, which turned out to be insignificant (*P*=.96). Consequently, the generated score was modeled using the background and years of experience, which also showed no significance (*P*=.65 and .61, respectively). Finally, the real score was integrated as a predictor, and background and experience were not shown be to significant factors, (*P*=.45 and .65, respectively); however, the real score was significant (*P<*.001). The generated score in relation to the real score and the final fitted regression model is illustrated in Figure 6.

**Figure 7:**
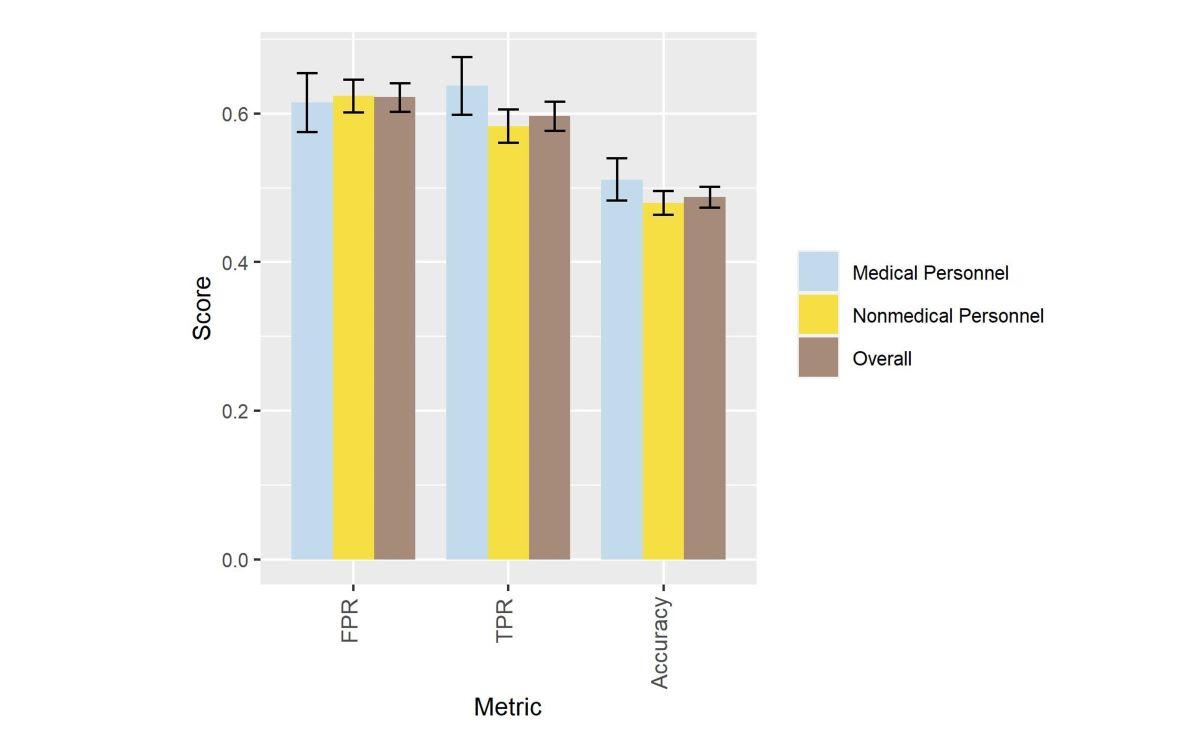
Evaluation of the human Visual Turing test results, with error bars representing 95% CI. FPR: false positive rate; TPR: true positive rate.

Consequently, we calculated the classification accuracy, FPR, and TPR to compare the generated images with the real ones. As illustrated in Figure 7, for all participating individuals regardless of background, the FPR was 0.62 (1511/2430 votes; 95% CI 0.60-0.64), and the TPR was 0.60 (1449/2430 votes; 95% CI 0.58-0.62), indicating high realism of the generated images. Moreover, there was no significant difference between the FPR of medical personnel and nonmedical personnel, which was 0.615 (95% CI 0.58-0.65) versus 0.624 (95% CI 0.60-0.65). The overall accuracy was 0.49 (95% CI 0.47-0.50), indicating that the participants had poor differentiation between generated and real images.

**Figure 8:**
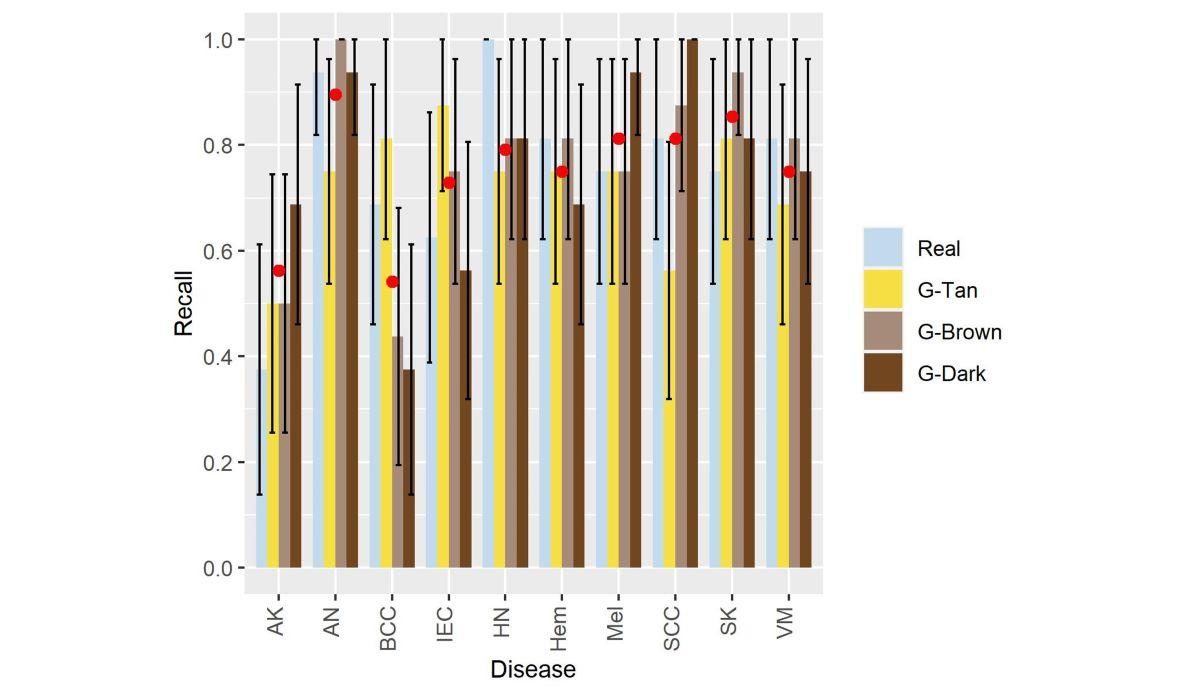
Recall of the utilized diseases, with error bars representing 95% CI. AK: actinic keratosis; AN: atypical nevi; BCC: basal cell carcinoma; IEC: intraepidermal carcinoma; HN: halo nevus; Hem: hemangioma; Mel: melanoma; SCC: squamous cell carcinoma; SK: seborrheic keratosis; VM: vascular malformation.

The second human qualitative assessment aimed to evaluate the accuracy of disease presentation in the generated images. We included a total of 80 images: 20 real images and 60 ST method–generated images (20 each for tan, brown, and black skin colors). The diseases included are shown in Figure 8. Eight expert dermatologists, masked to our study methodology and image sources, participated in a survey comprising real and generated images and chose a diagnosis most consistent with the image presented. The average recall (rate of correctly diagnosed lesions by dermatologists) of the real images was 0.76 (121 correct diagnoses out of 160) compared to 0.75 (360 correct diagnoses out of 480) for the generated images. Details of the recall for each disease group, image type, and skin color are demonstrated in Figure 8.

In Figure 8, the average recall of the generated images grouped by skin color, tan (G-Tan), brown (G-Brown), and dark (G-Dark), is represented by a red dot to compare to the real images. As this figure shows, basal cell carcinoma had the lowest average recall of the generated images compared to the real recall. In basal cell carcinoma, the tan-generated images had a recall of 0.81 compared to a real image recall of 0.69; however, the brown and dark images had a significantly lower recall of 0.44 and 0.38, respectively. Therefore, further analysis was performed to gain a deeper insight into the disease misdiagnosis.

**Figure 9:**
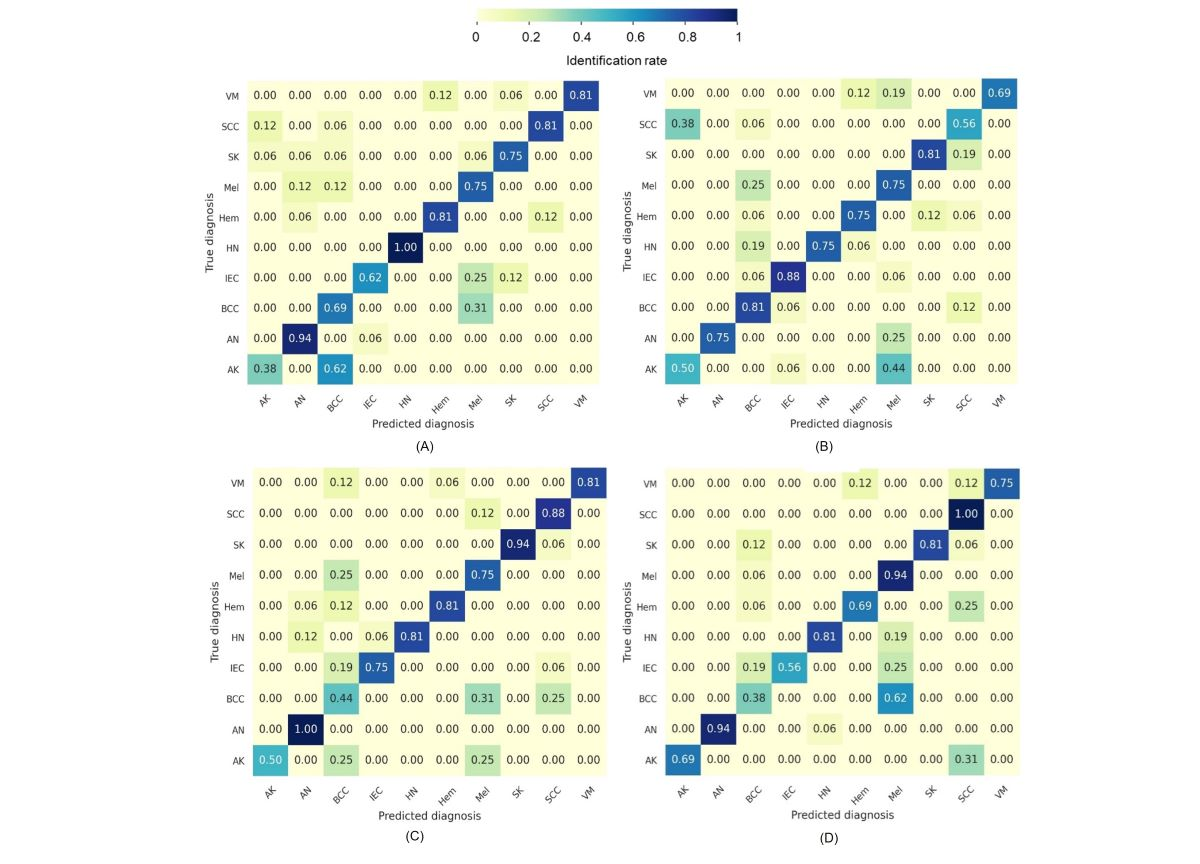
Confusion matrix of the real and generated images. (A) real images, (B) tan-generated images, (C) brown-generated images, and (D) dark-generated images.

The results of the recall experiment were summarized as confusion matrices for the real, generated tan, brown, and dark images, as shown in Figure 9A-D. The diagonal of the confusion matrix represents the rates of correctly diagnosed diseases (true positives), while all other numbers in the matrix represented the misdiagnosis rates.

It can be observed that basal cell carcinoma in the brown and dark skin images was mainly misdiagnosed as melanoma with a misidentification rate of 0.31 and 0.62, respectively. A closer look at the confusion matrix of the dark generated images (Figure 9D) reveals that intraepidermal carcinoma was also misdiagnosed as melanoma with a misidentification rate of 0.25. In addition, halo nevus was misidentified as melanoma with a rate of 0.19. On the other hand, melanoma was best identified in the dark skin color with a rate of 0.94. This high rate could be explained by the misdiagnosis of several lesions as melanoma. Thus, any pigmented lesion on the dark skin was primarily misdiagnosed as melanoma.

### Preliminary Classification Evaluation

A total of 4 models were developed: trained on set A images without augmentation (model 1), trained on set A augmented with the ST-generated images (model 2), trained on set A augmented with geometric transformations (eg, flipping, rotation, and noise) (model 3), and set A augmented with both the generated and transformed images (model 4). To assess the models’ generalizability, all were tested on set B, which entirely consisted of real images and was characterized by a different skin color distribution compared to the training set A (Table 2).

A comparison between the accuracy and AUC of the developed models is shown in Table 5. It can be observed that model 1 is the least performing model because it has the least discrimination ability characterized by the least AUC of 0.63. On the other hand, model 2 is the best performing model with an accuracy and AUC of 0.76 and 0.72, respectively, indicating the significant impact of the skin color augmentation on the model’s generalizability. With respect to model 3 (AUC 0.66), a comparable performance to model 1 (AUC 0.63) can be noticed, indicating that geometric transformations did not significantly increase the model’s performance. Finally, model 4 (AUC 0.69) showed improved performance compared to model 3 (AUC 0.66) but decreased performance compared to model 2 (AUC 0.72), emphasizing that combining several data augmentations did not benefit the model.

It can be concluded that augmenting the data with diverse skin color images allowed the model to learn skin tone–related features; thus, model 2 was robust to the variations of the skin color in the test set. On the other hand, the geometric transformations did not provide the model with the variability needed to handle the deviation in skin tone distribution present in the test set. Therefore, when combined with the generated images, a decrease in performance was noticed, highlighting the importance of selecting consistent image augmentations that work to fill the gap between the training and testing data [38].

Finally, to evaluate the significance of the difference in the AUC between the best performing model (model 2) and all other models, the Delong test to compare 2 ROC curves [46] was carried out. The difference in AUC between models 2 and 1 and between models 2 and 3 was significant (*P*<.001 and *P*=.03, respectively), while there was no significant difference in the AUC between models 2 and 4(*P*=.35).

**Table 5:** Performance of the classification models on set B.

Models	Accuracy	AUC^a^
Model 1	0.56 (95% CI 0.52-0.60)	0.63 (95% CI 0.58-0.68)
Model 2	0.76 (95% CI 0.72-0.79)	0.72 (95% CI 0.67-0.77)
Model 3	0.56 (95% CI 0.52-0.60)	0.66 (95% CI 0.62-0.71)
Model 4	0.60 (95% CI 0.56-0.64)	0.69 (95% CI 0.65-0.74)

^a^AUC: area under the curve.

## Discussion

### Principal Results

In this work, we proposed a DL-based approach to generate realistic skin images for underrepresented skin colors using publicly available white skin clinical images. We utilized the pathology of light skin images and healthy dark skin images to extract and blend disease and pigmentation features. The employed strategy of generating darker images based on feature blending helped to overcome the lack of dark skin images, as the utilized image generation techniques herein were trained to extract high-level features from images independently from their content [26]. In terms of evaluating the quality of the generated images, comprehensive qualitative and quantitative approaches were developed. Given that the qualitative analyses can be affected by the paucity of darker skin images and because human judgment (especially the disease diagnoses test) might vary based on skin color, we performed statistical and mathematical quantitative analyses to address this issue. The results emphasized that ST-generated images had high realism and disease presentation, characterized by a lower loss of realism and higher structural similarity scores for all skin colors compared to those based on the DB method. Moreover, the generated images achieved high FPR and disease recall when compared to the real images. Finally, the generated images contributed to improvement in the classification performance when used to augment the training of ResNet-50 in comparison to other augmentation strategies.

### Limitations

Our work has several noteworthy limitations and areas for future improvement. Lesion pigmentation is not the only factor that characterizes skin cancer in people of color; thus, other disease morphological features need to be integrated into our models. As such, in Phase 4, text features representing skin cancer clinical presentation on darker skin will be created based on the published literature and consequently utilized along with the augmented images to train the classification models. In addition, the classification accuracy that has been investigated herein needs to be improved; therefore, in Phase 4, several CCN architectures and ensemble learning methods will be implemented to boost the classification accuracy. Moreover, images with real pathology in people of color are required to improve model training and validation. Finally, it is worth mentioning that other novel skin tone scales have been recently developed, such as Google’s Monk scale [47]. Thus, our skin tone categorization tool can benefit from investigating and validating such new scales.

### Comparison With Prior Work

Image generation using DL has been applied in the literature to improve data balance. The generative adversarial network (GAN) has been utilized to generate synthetic images for several malignant and benign lesions to overcome class imbalance [48]. The model was trained on 10,000 dermoscopic images from the ISIC-2018 data set, and the generated images were evaluated for realism by humans. A total of 3 dermatologists and 5 DL experts classified a random sample of the real and generated images as real or fake. The analysis showed that the human classification accuracy was around 50%, meaning that the raters were not able to clearly distinguish between real and generated images. However, generating images with various skin colors was not considered in the aforementioned study.

GAN was also employed to generate dermoscopic images to mitigate data imbalance. Three GAN models were trained on 2000 dermoscopic images from the ISIC-2017 data set [49]. To evaluate the generated images, the authors compared the normalized color histogram of the generated images with the training images. Their results showed a high similarity in the distribution of both real and generated images. Despite the high quality of the generated images, there was no focus on skin color.

In another study [50], the authors utilized GAN to generate clinical skin images for various skin conditions, in which the required input features (eg, skin color and lesion location) were manually encoded. Encoding of input features was required during all model development phases (eg, training, validation, and testing); thus, the developed model could not be deployed without feature encoding. Although the images could be generated with different skin colors using the encoding maps, no images were generated with dark skin colors.

In terms of evaluation, the realism of the generated images in the aforementioned study [50] was evaluated by conducting a VTT with 10 participants, and the generated images had an average FPR of 0.3. Meanwhile, in our work, the VTT was conducted with 54 participants and achieved a higher FPR of 0.62. Moreover, the disease recall evaluation was conducted with 2 dermatologists and achieved an average recall of 0.45. However, in our work, the disease recall was assessed with 8 dermatologists and achieved a significantly higher average recall of 0.75. Furthermore, we performed a misdiagnosis analysis, and our findings strongly agreed with the published literature on skin cancer misdiagnosis in people of color [51].

### Conclusion

Despite the recent advances of AI in dermatology diagnosis, the lack of skin color diversity when training AI models is a major pitfall. Until a sufficient real-world diverse image repository is collected, augmenting real images with generated darker skin images is the first step to implementing robust diagnosis models. The generated images in this work achieved high realism and disease recall scores when compared to the real images. In addition, the generated images augmented the publicly available white skin images, and a classification model was developed that outperformed the model trained without the generated images. In our future work, which will comprise Phase 4 of this study, we will focus on overcoming our previously mentioned limitations to boost the accuracy and robustness of the preliminary classification model discussed herein. After completing all study phases and addressing all discussed limitations, the resulting model will be a tool to aid general practitioners in diagnosing possible skin malignancy and thereby improve the efficiency and reduce the redundancy of referrals that expert dermatologists receive for further clinical assessments and biopsies.

## Appendix

Multimedia Appendix 1 Image generation fine-tuning.

Multimedia Appendix 2 Individual typology angle.

Multimedia Appendix 3 Quantitative evaluation details.

## Abbreviations

AI: artificial intelligence
AUC: area under the curve
BRISQUE: blind referenceless image spatial quality evaluator
CNN: convolutional neural network
DB: deep blending
DL: deep learning
FPR: false positive rate
GAN: generative adversarial network
ISIC: International Skin Imaging Collaboration
ITA: individual typology angle
NSERC: Natural Sciences and Engineering Research Council of Canada
SSIM: structural similarity index measure
ST: style transfer
TPR: true positive rate
VGG: visual geometry group
VTT: visual Turing test
