# Supplementary material for: Improving Skin Color Diversity in Cancer Detection: Deep Learning Approach
Source: JMIR Dermatol. 2022 Aug 19;5(3):e39143. doi: 10.2196/39143 (PMC10334920; doi:10.2196/39143)
Supplement: Multimedia Appendix 1 [file derma_v5i3e39143_app1.pdf]

Image Generation Finetuning

In the ST image generation method, the parameters of the ST method were finetuned to fit the skin images. The process of generating dark skin images required 1,000 iterations to reach a stable state with no improvement in the total loss. For all tuning illustrations (Figure S1–3), the content image was a white-skinned basal cell carcinoma, and the style image was dark healthy skin. All the tunings were executed on several images while considering the recommended tuning values in the original work [24].

The content to style weight ratio R was tuned to control the dominance of the content and style features in the generated images. Higher values of R resulted in more content and less style features in the generated image, but with the decrease of R, the color and texture of the style were more present as illustrated in Figure S1. With R less than 0·001, the image started to show distortions, thus R=0·001 was selected. The depth of the VGG network for content and style feature extraction was tuned by changing the number of layers to control the representation level of the features extracted as shown in Figure S2. Layers L_4 and L_5 were chosen for the content and style respectively as they resulted in seamless features blending.

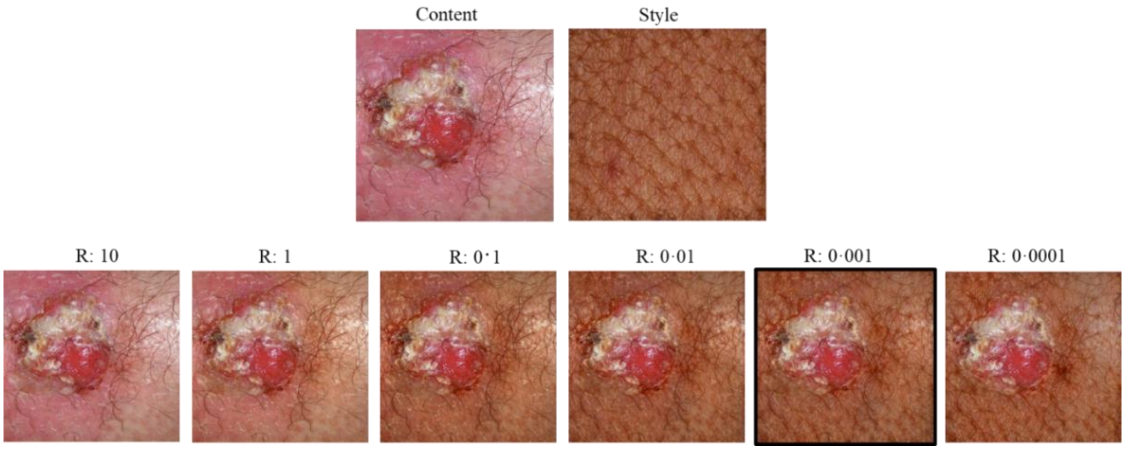

Figure S1. Content to style weight ratio tuning

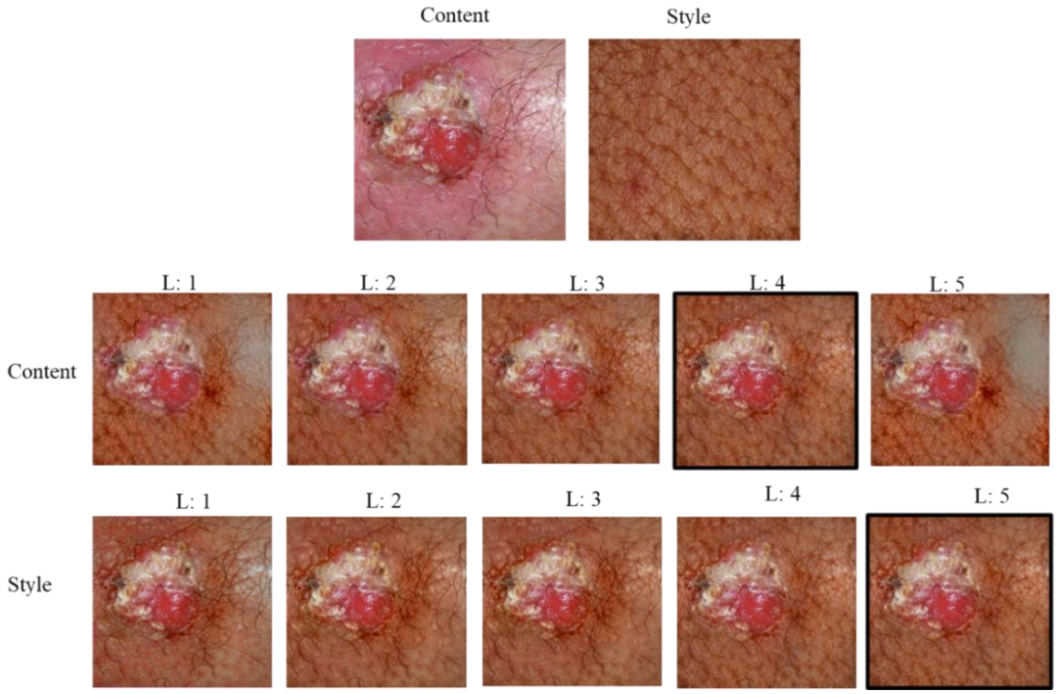

Figure S2. Content and style layers depth tuning

The style weight distribution between the disease region and the non-infected skin was adjusted to control the pigmentation and texture of the disease and improve image naturalness. In Figure S3, the style weight was adjusted from (0·5, 0·5) to (0,1). With a style weight of (0.5, 0.5), the style features were equally distributed among the disease and skin areas. However, with a style weight distribution of (0, 1), the style effect was completely removed from the disease region and focused on the skin. The selected weight distribution was (0·01, 0·99)—ensuring a visual balance between disease pigmentation and clarity.

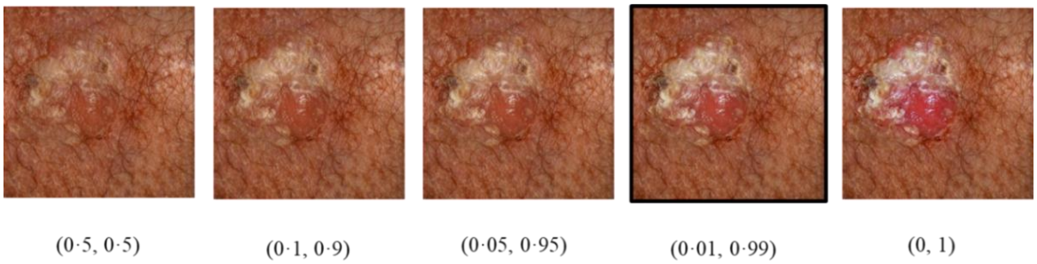

Figure S3. Style weight distribution

In the DB image generation method, two rounds of image optimization were performed, each round needed 1,000 iterations to generate the images and reach a stable total loss. Moreover, the content images were segmented using our augmented segmentation model discussed in [21] to separate the disease region from the skin. Consequently, the VGG network parameters were fine-tuned to fit the skin clinical images. The content to style weight ratio R ranged from 10 to 0·0001 as illustrated in

Figure S4. It can be noticed that the style skin image was dominant in all generated images and only the segmented disease region was the semantic feature set extracted from the content image. Therefore, the increase in R led to a richer view of the disease region, and thus R=10 was selected. There was no noticeable change in the generated image for R values greater than 10.

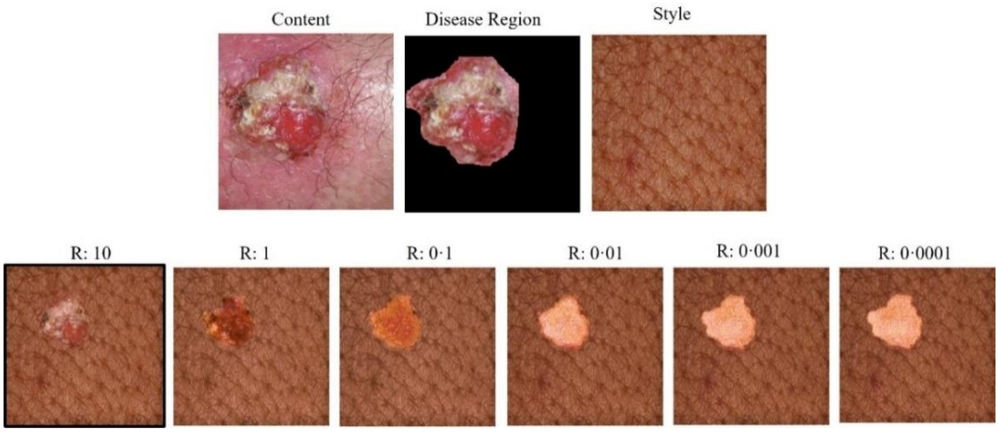

Figure S4. Deep blending content to style weight ratio I tuning

The VGG network layers were finetuned as shown in Figure S5, deeper content network created distortions in the disease region, therefore L_2 was selected for content. For the style network, increasing the layers resulted in a clearer disease region, therefore all style layers were included.

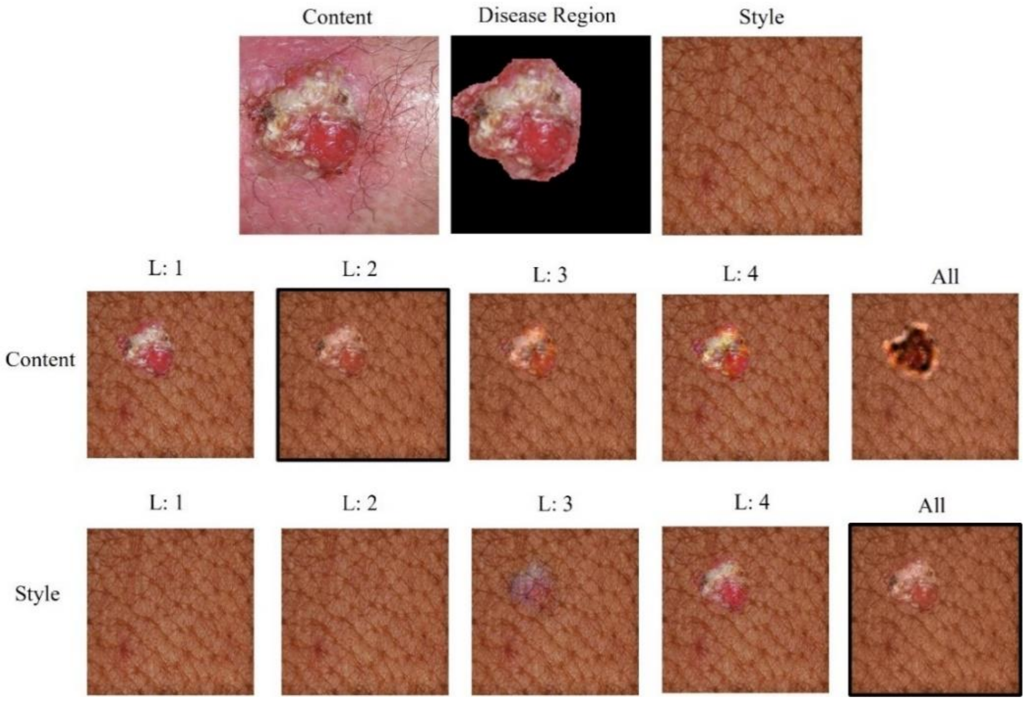

Figure S5. Deep blending content and style network layer (L) tuning
