# Supplementary material for: Improving Skin Color Diversity in Cancer Detection: Deep Learning Approach
Source: JMIR Dermatol. 2022 Aug 19;5(3):e39143. doi: 10.2196/39143 (PMC10334920; doi:10.2196/39143)
Supplement: Multimedia Appendix 2 [file derma_v5i3e39143_app2.pdf]

Individual Typology Angel

In the target skin color selection, skin images were processed to transform the values of the pixels from the default color space, RGB (red, green, and blue), to the CIE-Lab color space to obtain the L and b components. The L measures luminance and b measures the degree of yellowness in each pixel [29], consequently, the ITA was calculated as in Equation S1 [20]. Based on the calculated angle, skin images were classified as per the ranges defined in Table S1 [30].

$$ITA = arctan(\frac{L-50}{b}) \times \frac{180°}{\pi} \qquad (Equation\ S1)$$

Table S1. Skin color classification according to the ITA

ITA	Skin Classification
>55°	Very light
41° to <55°	Light
28° to <41°	Intermediate
10° to <28°	Tan
-30° to <10°	Brown
<-30°	Black
