# Supplementary material for: Improving Skin Color Diversity in Cancer Detection: Deep Learning Approach
Source: JMIR Dermatol. 2022 Aug 19;5(3):e39143. doi: 10.2196/39143 (PMC10334920; doi:10.2196/39143)
Supplement: Multimedia Appendix 3 [file derma_v5i3e39143_app3.pdf]

Quantitative Evaluation Details

In the BRISQUE quantitative image evaluation metric, the mean subtracted contrast normalized coefficients of each pixel represented as $\hat{I}(i,j)$ and its neighboring pixels including the horizontal *H(i,j)*, vertical *V(i,j)*, diagonal $I_1(i,j)$, and off-diagonal $D_2(i,j)$ neighbors (Figure S1) are calculated (Equations S1–5).

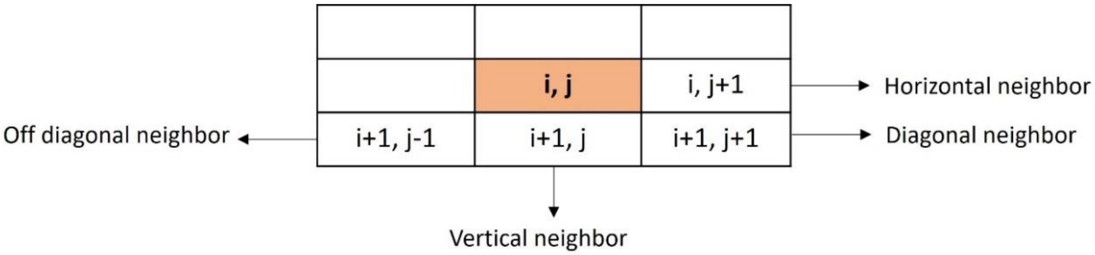

Figure S1. The neighbors of a target pixel

$$\hat{I}(i,j) = \frac{I(i,j) - \mu(i,j)}{\sigma(i,j) + C} \quad \text{(Equation S1)}$$

$$H(i,j) = \hat{I}(i,j)\hat{I}(i,j+1) \quad \text{(Equation S2)}$$

$$V(i,j) = \hat{I}(i,j)\hat{I}(i+1,j) \quad \text{(Equation S3)}$$

$$D_1(i,j) = \hat{I}(i,j)\hat{I}(i+1,j+1) \quad \text{(Equation S4)}$$

$$D_2(i,j) = \hat{I}(i,j)\hat{I}(i+1,j-1) \quad \text{(Equation S5)}$$

where *I(i,j)* is the intensity of the pixel at positIons *i, j*. μ is the local mean and σ is the standard deviation estimated using a weighting Gaussian function. C is a constant to avoid the instability of the calculations when the denominator approaches zero.

The generalized Gaussian distribution [33] and the asymmetric generalized Gaussian distribution models [34] are employed to estimate the mean and the standard deviation. Consequently, the calculated metrics are passed to a regression model to assign a quality score for the image.

In the SSIM image evaluation metric, to calculate the SSIM score, each image is divided into smaller windows with the same spatial positions in the modified and reference images. Consequently, the average pixel values, standard deviation, and covariance for each window in both images are calculated as shown in Equations S6–9.

$$l(x,y) = \frac{2\mu_x\mu_y + C_1}{\mu_x^2 + \mu_y^2 + C_1} \quad \text{(Equation S6)}$$

$$c(x,y) = \frac{2\sigma_x\sigma_y + C_2}{\sigma_x^2 + \sigma_y^2 + C_2} \quad \text{(Equation S7)}$$

$$r(x,y) = \frac{\sigma_{xy} + C_3}{\sigma_x \sigma_y + C_3} \quad \text{(Equation S8)}$$

$$SSIM(x,y) = l(x,y)\,.\,c(x,y)\,.\,r(x,y) \quad \text{(Equation S9)}$$

where x and y are equal-sized windows from two images X and Y. Average pixel values μ_x, μ_y to estimate brightness differences, standard deviation pixel values $\sigma_x \sigma_y$ to estimate the contrast differences, and covariance σ_{xy} to estimate structure differences. Finally, $C_1, C_2,$ and C_3 are constants to avoid the instability of the calculations when the denominators approach zero.
